# Prehospital Double Defibrillation for Refractory Ventricular Fibrillation: A Scoping Review Protocol

**DOI:** 10.19102/icrm.2020.110603

**Published:** 2020-06-15

**Authors:** Dennis Miraglia, Lourdes A. Miguel

**Affiliations:** ^1^Department of Internal Medicine, Good Samaritan Hospital, Aguadilla, Puerto Rico

**Keywords:** Double defibrillation, out-of-hospital cardiac arrest, refractory ventricular fibrillation

## Abstract

Double defibrillation (DD) has been proposed as an alternative treatment for patients with refractory ventricular fibrillation/pulseless ventricular tachycardia (VF/pVT) out-of-hospital cardiac arrest (OHCA) nonresponsive to the best current standard of care. Treatment results are promising, but the efficacy and safety of the procedure remain unclear. Currently, there is a paucity of evidence in the literature on DD suggesting the optimal strategy for treating this challenging patient population. Thus, we aim to perform a scoping review to explore the current literature addressing resuscitative parameters, survival rates, and neurological outcomes in refractory VF/pVT OHCA patients treated with DD as well as to identify gaps in the literature that may require further research. Here, we discuss the anticipated study protocol.

## Introduction

### Background

Survival to hospital discharge and neurologically intact survival from refractory ventricular fibrillation/pulseless ventricular tachycardia (VF/pVT) out-of-hospital cardiac arrest (OHCA) has a dismal prognosis even when patients are treated by highly specialized first-responder units.^1–3^ Data from the Resuscitation Outcomes Consortium (ROC) in 2016 suggest the incidence of OHCA is 110.8 per 100,000 people or 347,000 adults annually in the United States. More broadly, OHCA remains a leading cause of death worldwide, accounting for up to one death per 1,000 people.^4,5^

Double defibrillation (DD) has been proposed as an alternative treatment for patients with refractory VF/pVT OHCA nonresponsive to the best current standard of care.^6^ However, the efficacy and safety of the procedure remain unclear at this time. DD involves the use of two defibrillators to deliver the maximum allowed energy that may be necessary to treat refractory VF/pVT. DD treatment deployment may either be sequential or simultaneous, depending on the duration of the defibrillation potential and the intershock interval between the two defibrillator shocks. When applying DD, one set of pads is placed in the anteroposterior position and another set of pads is placed in the anterolateral position.^7–9^ There is currently no scientific evidence regarding the exact mechanism of DD for prehospital refractory VF or any evidence suggesting that the sequential or simultaneous method is more effective. Nevertheless, there are a few theories as to why DD is effective.

### Study rationale

To improve outcomes of refractory VF/pVT OHCA, some researchers have previously looked at registry data to describe their experience with DD. Further, different protocols with no uniform guidelines and a variety of consensus papers have also been published.^10–16^ Whether these studies are based on the best available evidence remain uncertain and this has been questioned in the medical literature. Also, results of prehospital DD for refractory VF/pVT are promising but still inconclusive.^11–13,17–20^ The right DD protocol for refractory VF/pVT OHCA seems to be heterogenic. Currently, there are no uniform guidelines or consenses for when DD should be applied to a patient who remains in refractory VF/pVT OHCA. Case reports and case series on prehospital DD have described inconsistent approaches^10,12,19^; similarly, there were variations reported in the approaches of observational studies.^13–16^ Heterogenic implementation of timing to DD may lead to suboptimal reanimation results and may affect both efficacy and survival outcomes in patients with refractory VF/pVT OHCA.

### Objectives

Given the above discrepancies, it was thought essential to systematically review the literature addressing the efficacy of DD in patients with refractory VF/pVT OHCA as well as their survival rates and neurological outcomes. The planned scoping review will address (1) what is known currently from the literature about the efficacy of DD for patients in refractory VF/pVT OHCA, (2) what are the survival rates and neurological outcomes among these patients after survival to discharge, and (3) what are the implications of this protocol for these patients and the potential gaps in this field?

## Methods and analysis

This scoping review protocol was written based on the principles outlined by the Preferred Reporting Items for Systematic Review and Meta-analysis Protocols (PRISMA-P),^21^ which define the following methodology upon which the scoping review will be based. To this end, literature searches and critical assessments will be performed.

### Eligibility criteria

We will use the Population, Concept, and Context framework to delineate eligibility criteria.^22^ We will include any controlled clinical study designs (randomized controlled trials and nonrandomized controlled trials) and observational studies (cohort and case–control studies) with a control group (i.e., patients not receiving DD) that provide information on resuscitative parameters, survival rates, and neurological outcomes in adults (> 18 years) treated with DD with the need for resuscitation due to refractory VF/pVT OHCA. Abstracts, case reports, case series, duplicate studies, and reviews will be excluded. Articles that do clearly distinguish refractory VF/pVT OHCA, those written in languages other than English, or those that include pediatric populations or pregnancies will also be excluded.

### Information sources

Search terms will combine controlled terms and free-text searches **(Table 1)**. The search strategies will be adapted to each electronic database. To identify relevant research, we will conduct searches from the beginning of DD for refractory VF/pVT OHCA in the electronic databases Medline, Embase, Scopus and Google Scholar. We will also review the references of relevant articles and perform a comprehensive cited reference search. The search will be repeated one month after the start of the review process to capture any articles published since its commencement. The language of articles eligible for inclusion will be restricted to English. Owing to the main research question, we decided to conduct a broad search, as including more specific search terms would, in our case, be associated with a higher risk of missing incorrectly labelled studies in the literature. Further, to identify ongoing clinical trials, we will search the International Clinical Trials Registry Platform (http://www.who.int/ictrp/en/), which includes entries in ClinicalTrials.gov.

For the database search, the Medline literature search terms used will be as follows: “cardiopulmonary resuscitation” or “CPR” or “cardiac arrest” or “heart arrest” or “advanced cardiac life support” or “ACLS” or “dual defibrillation” or “double defibrillation” or “double simultaneous defibrillation” or “double sequential defibrillation” or “DD” or “DSD” or “out-of-hospital cardiac arrest” or “OHCA” or “prehospital cardiac arrest” or “ventricular fibrillation” or “VF” or “ventricular dysrhythmia” or “refractory ventricular fibrillation” or “RVF.”

### Review process

Following retrieval of all identified studies and the removal of duplicate publications, the selected studies will be screened by two independent reviewers, first based on their title and abstract and, subsequently, based on their full text. Following the identification of eligible studies in this manner, we will extract the required information into a predefined standardized form that will be piloted on two articles. All disagreements will be resolved by the addition of a third author. The methodology of this scoping review may be adapted minimally during the review process itself with regard to eligibility criteria, study characteristics, and outcome variables. A risk-of-bias assessment will not be part of the methodology of the scoping review.^23,24^

### Data items

Data will be extracted in standardized tables that will be set up in a predefined standardized form in Microsoft Excel 2019 version 16.26.1 (Microsoft, Redmond, WA, USA). Variables extracted for data charting will include the first author, year of publication, study design, focus of the paper, population sample size, population characteristics, interventions, key outcomes, and limitations **(Table 2)**. The details of resuscitative parameters may be extended during the review process.

### Outcomes

Outcomes variables are listed in **Table 2**.

### Data synthesis

We expect to observe dramatically heterogeneous study characteristics within the field of DD. This may express itself in terms of the patient population and DD procedure as well as the protocol. Therefore, as one of the first steps of our scoping review of the literature, we will summarize the gathered data using figures and tables to present the research and describe potential gaps in this field.

Detailed results will be presented in two tables. One table will present the characteristics of patients in refractory VF arrest before receiving DD, while another table will present the characteristics of patients in refractory VF arrest after receiving DD and subsequent outcomes. In both tables, studies will be listed chronologically, beginning with the latest publication. In addition to the tabulated presentation of data, each study outcome will be addressed and discussed separately in the study’s main text.

### Strengths and limitations

The scoping review will be reported according to the PRISMA-P statement for scoping reviews. Using a computerized, structured data abstraction form, two members will independently conduct the study inclusion and data extraction processes to avoid bias during the research process. To avoid common heterogeneity of the outcomes presentation, we predefined the outcome variables, which will be incorporated in the scoping review. Overall, we aim to perform a comprehensive literature review addressing DD for refractory VF/pVT OHCA. The data presented will be consistent, precise, and relatively easy to analyze. Unfortunately, most studies in prehospital care deal with incomplete data and are not robust enough, which is especially so for studies on DD for refractory VF/pVT; hence, heterogeneity may be a significant issue.

### Research versus real clinical application

DD is currently being employed in a number of Emergency Medical Services (EMS) systems across the United States. However, previous research in the prehospital refractory VF population has been limited by the omission of data regarding the time from cardiac arrest to DD, the lack of consensus regarding a standard definition of refractory VF, and variations in EMS protocols. For the purpose of moving forward with additional studies on DD, this scoping review protocol will facilitate the opportunity for discussion on DD and evaluate both the implications of this therapy for prehospital cardiac arrest and the potential gaps remaining in this field. Further, it is necessary to agree upon a uniform definition of refractory VF and create a standardized protocol for the treatment of prehospital VF that can easily be implemented by EMS providers.

We propose such a definition and protocol below based on the potential benefits and on the basis of available evidence. At the time of EMS arrival, advanced cardiovascular support guidelines should be applied for prehospital VF. Patients with prehospital cardiac arrest and a diagnosis of refractory VF who do not respond to at least three defibrillation attempts, 3 mg of epinephrine, and/or 300 mg of amiodarone and who remain in VF should be prepared for the fourth shock and the second set of pads should be placed. Both defibrillators should be set at the maximum energy to charge and a single operator should push the shock buttons on each machine as simultaneous as possible. However, the issue of the efficacy of DD may not be an energy problem; instead, it may simply be the fault of an alternate plane or vector of defibrillation established by how the pads are placed.^16^ If the initial pads are placed in the anterolateral position, one should place the second set of pads in the anterioposterior position and vice versa.

Studies on patients resuscitated from VF/pVT cardiac arrest have demonstrated that these patients have significant coronary stenosis in up to 50% of the cases^25–27^ and that coronary artery disease is the most common reversible underlying cause.^28,30^ Therefore, these patients will benefit from first-responder units being able to provide automated cardiopulmonary resuscitation with a LUCAS device (Physio-Control Corp., Redmond, WA, USA), impedance threshold device (ResQPOD™; ZOLL Medical Corporation, Chelmsford, MA, USA), and earlier deployment of venoarterial extracorporeal membrane oxygenation-assisted revascularization as a bridge to definitive treatment.^29–31^

### Perspective

If the scoping review provides us with enough study data, we plan to conduct a subsequent systematic review and meta-analysis to evaluate the efficacy of DD in patients with refractory VF/pVT OHCA.

### Dissemination

We intend to publish the scoping review in a peer-reviewed journal.

## Figures and Tables

**Table 1:** Summary of Search Terms

No.	Search Items (Controlled Terms)
1	Cardiopulmonary resuscitation
2	CPR
3	Cardiac arrest
4	Heart arrest
5	Advanced cardiac life support
6	ACLS
7	Dual defibrillation
8	Double defibrillation
9	Double simultaneous defibrillation
10	Double sequential defibrillation
11	DD
12	DSD
13	Out-of-hospital cardiac arrest
14	OHCA
15	Prehospital cardiac arrest
16	Ventricular fibrillation
17	VF
18	Ventricular dysrhythmia
19	Refractory ventricular fibrillation
20	RVF

ACLS: advanced cardiovascular support; CPR: cardiopulmonary resuscitation; DD: double defibrillation; DSD: double sequential defibrillation/double simultaneous defibrillation; OHCA: out-of-hospital cardiac arrest; RVF: refractory ventricular fibrillation; VF: ventricular fibrillation.

**Table 2:** Study Characteristics and Outcomes

Type of Information	No.	Data Extraction (Selection and Coding)	
Study characteristics	1	–	First author
		–	Year of publication
		–	Country of the study
		–	Study design
		–	Number of patients screened
		–	Number of patients included
Patient characteristics	2	–	Population studied (eg, age, comorbidities, presumed or confirmed cause of cardiac arrest, location of cardiac arrest)
Intervention characteristics	3	–	Number of standard defibrillations performed
		–	Number of double defibrillations performed
		–	Number of patients with available outcomes data
		–	Type of ventricular dysrhythmia (VF/pVT)
		–	Initial or subsequent ventricular dysrhythmia
		–	Defibrillator characteristics (monophasic or biphasic, manual or automatic)
		–	Energy dose used for the first shock
		–	Energy dose used for double defibrillations shocks
		–	Total energy dose
		–	Pad placement (ie, anterolateral, anteroposterior)
		–	Time from collapse to double defibrillation
		–	Number of double defibrillations attempts to VF termination
		–	Number of double defibrillations performed to VF termination (ie, ROSC, PEA, asystole)
		–	Number of double defibrillations performed to sustained ROSC
		–	Resuscitation time
Exclusion of the study based on intervention characteristics	4	–	Nonadherence to eligibility criteria
		–	No information given on resuscitative parameters
Outcome variables	5	–	VF termination into sustained ROSC
		–	Sustained ROSC
		–	Survival to hospital admission
		–	Survival to hospital ICU admission
		–	Survival rate to hospital discharge, 30 days, 3 months, 6 months, and long-term
		–	Favorable neurological outcome to hospital discharge, 30 days, 3 months, 6 months, and long-term
Exclusion of the study based on outcome variables	6	–	No information given on survival rate or neurological outcome
		–	No information given on time frame of survival rate
		–	No information given on time frame of favorable neurological outcome

ICU: intensive care unit; PEA: pulseless electrical activity; ROSC: return of spontaneous circulation; pVT: pulseless ventricular tachycardia; VF: ventricular fibrillation.
